# Thallium in spawn, juveniles, and adult common toads (*Bufo bufo*) living in the vicinity of a zinc-mining complex, Poland

**DOI:** 10.1007/s10661-014-4141-7

**Published:** 2014-11-25

**Authors:** Krzysztof Dmowski, Monika Rossa, Joanna Kowalska, Beata Krasnodębska-Ostręga

**Affiliations:** 1 Department of Ecology, Faculty of Biology, Biological and Chemical Research Centre, University of Warsaw, Żwirki i Wigury 101, 02-089 Warsaw, Poland; 2 Faculty of Chemistry, University of Warsaw, Pasteura 1, 02-093 Warsaw, Poland

**Keywords:** Thallium, Pollution, Toads, Trace elements, Amphibians, Maternal transfer

## Abstract

A breeding population of the common toad *Bufo bufo* living in the vicinity of a Zn-Pb smelting works in Bukowno, Poland was studied for the presence of thallium. Tl concentration was measured in the bottom sediments of the spawning pond, in the laid eggs, in juveniles after metamorphosis, and in the selected tissues of the adult individuals. A very high concentration of Tl was detected in the spawn (13.97 ± 8.90 mg/kg d.w.). In 50 % of the spawn samples, levels exceeded 20 mgTl/kg d.w. The issue of maternal transfer of thallium from females to oocytes is discussed. Due to a significant accumulation of thallium, spawn analysis can be used as a sensitive indicator of the presence of this element in the environment and may replace more invasive methods that involve the killing of adult animals. In those regions that are abundant in Zn-Pb ores, the spawn of amphibians may be a very important source of thallium contamination for predators. From among all tissues of the Bukowno adult toads, the livers have shown the highest accumulation of thallium (mean 3.98 mg/kg d.w. and maximum value—18.63). For as many as 96.5 % of livers, concentrations exceeded 1.0 mgTl/kg d.w. which is treated as indicative of poisoning.

## Introduction

Thallium is a very toxic element, recognized by the Environmental Protection Agency as a xenobiotic that is even more dangerous than mercury and lead. Thallium was used extensively since the 1920s in the production of rat poisons and insecticides. Since the 1970s, a ban on thallium-laced pesticides has been issued as per recommendations by the World Health Organization (WHO 1996; Nriagu 1998).

On the molecular level, thallium poisoning involves the impairment of the central and peripheral nervous system as well as the digestive and the circulatory systems. The main reason why thallium is so toxic is its tendency to replace the K + ion in numerous biochemical reactions. Thallium then influences such fundamental physiological processes as neurotransmission and the activation of the muscle cells or inhibits the functioning of important enzymes, for example kinases. Tl causes irreversible damage to ribosomes and the mitochondria (Manzo and Sabbioni 1988; Kelner 1994; Sager 1994; Galvan–Arzate and Santamaria 1998; Nriagu 1998; Peter and Viraraghavan 2005).

**Fig. 1:**
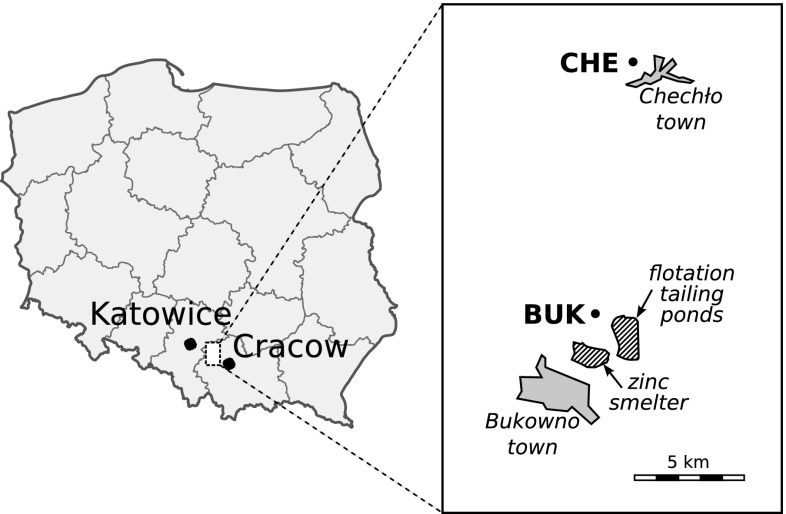
Location of study areas

The most anthropogenic sources of thallium in the environment are mining and the smelting of sulfidic metal ores during Zn-Pb processing (Ewers 1988; WHO 1996; Kazantzis 2000). In Poland, the highest emissions of thallium have been recorded in the vicinity of a Zn-Pb smelting and mining complex in the Bukowno-Olkusz region between the towns of Katowice and Cracow (Fig. 1). Post-flotation wastes are gathered here in ponds atop large raised heaps of tailings that are piled up to heights of 20–40 m. Initially, four tailing ponds stretched over an area of roughly 110 ha, at present just one active 37-ha pond remains. Aside from the main ore minerals (Zn, Pb, and Fe), such additional trace elements as Ag, Cd, Mn, Ni, Se, and the extremely toxic Tl have been detected in the tailing ponds (Kucha and Jędrzejczyk 1995; Lis et al. 2003; Cabała et al. 2004, 2009). The amount of thallium that still remains in the inactive tailing ponds is estimated at 665 tonnes (Górecka and Bellok 1998). The drying wastes are easily dispersed in the form of dust particles within the area of the smelting complex, thus increasing the concentration of thallium in the upper layers of the soil. Soil samples (Helios-Rybicka et al. 2004; Karbowska et al. 2003; Jakubowska et al. 2007) that were collected in the direct vicinity of the flotation tailing ponds have been found to contain as much as 5–50 mg/kg d.w. of thallium and amounts of 0.48–3.46 mg/kg were found in grasses (Adamiec and Helios-Rybicka 2004). The highest concentration of thallium in local plants was indicated by Wierzbicka et al. (2004) in the leaves of ribwort plantain (*Plantago lanceolata*)*—*54 mg/kg d.w.

Tl concentration in unpolluted plant and animal samples are in the scope 0.00 × –0.0× mg/kg while values equal to or bigger than 1.0 mg/kg d. w. are treated as a sign of thallium contamination (Manzo and Sabbioni 1988; Nriagu 1998; Kabata-Pendias and Mukherjee 2007). Thallium contents in the kidneys and livers of Danish birds of prey exceeding 2 mg/kg d.w. were believed to be indicative of poisoning (Clausen and Karlog 1977). Natural concentrations of thallium in plants usually range between 0.01 and 0.3.mg/kg. Natural thallium levels in human organs are generally in the scope of <0.001–0.005 mg/kg f.w., and rarely exceed 0.010 mg/kg. (Manzo and Sabbioni 1988) while in the organs of livestock are in the scope 0.07–0.33 mg/kg d.w. (Kemper and Bertram 1991). Similar or lower levels of Tl in tissues of European farm mammals and fishes have been reported int. al. by the WHO (1996) and Das et al. (2006).

Data for thallium contamination of the fauna living in the Bukowno-Olkusz region are very scant. Studies by Dmowski (2000) on magpies and Dmowski et al. (1998) on rodents fully confirmed contamination with this element. Native frogs and toads have never been tested for the presence of thallium in tissues, in spite of significant contamination of local water bodies, including those reservoirs that serve as their breeding areas.

The aim of the present study was to determine concentrations of thallium in the spawn, juveniles after metamorphosis, and in major organs of adult common toads (*Bufo bufo*) living in the vicinity of flotation tailing heaps in Bukowno. Since the chemical analysis of the tadpoles would require the removal of the digestive tract and its highly contaminated content directly at location (in situ), such analysis—due to the small size of the larvae and the likelihood of accidental contamination of the samples—was not performed.

## Material and methods

### Studied species

The common toad (*B. bufo)* was chosen because it has a short breeding season and enters bodies of water for a relatively short period of time (a few days for the females, slightly longer for the males). Eggs are laid in a chain form, in masses during one joint event with no further re-depositions over the subsequent weeks; therefore, it is safe to assume that eggs collected from various locations of the reservoir were of the same age (Juszczyk 1987).

### Study areas

The main research site—pond Bukowno (BUK)—was a post-mining depression (size ∼7500 m^2^) filled with water. This pond was located at a distance of circa 800 m from the edge of the post-flotation heap, and circa 1500 m from the smelting works in Bukowno (Fig. 1). Pond Chechło (CHE) located near a town of Chechło, at a distance of circa 11 km north from the contaminated areas, was chosen as the control site. The distance to the BUK site was relatively close, but geological conditions were different with a significantly lower impact of industrial emissions. Pond CHE was a man-made reservoir, ∼2000 m^2^ in size, with a nearby spring as the main source of its water supply.

Since data as per concentrations of Tl around the two reservoirs were not available, levels of lead and cadmium were used as indicators of pollution with metallic xenobiotics. According to Lis and Pasieczna (1995), lead concentrations in the bottom sediment of the water bodies located at the BUK region ranged between 3200 and 6400 mg/kg d.w. For the CHE site, these concentrations did not exceed levels of 100–200 mg/kg d.w. Concentrations of Cd in BUK and CHE sediments reached 25–50 mg/kg and 3–6 mg/kg d.w., respectively.

### Sampling

Biological samples were taken from the ponds BUK and CHE. One-day-old eggs were collected from 10 different oviposition sites, and one sub-sample consisted of a dozen eggs. Egg masses and juveniles (8 sub-samples, each consisting of 8–9 individuals) were placed in sterile, conditioned (cleaned with 3–5 % HNO_3_) plastic 25-ml containers.

Twenty nine adult individuals from the BUK pond and 20 individuals from the CHE pond were collected in the spring of year 2005. Thallium concentrations were measured in the livers, kidneys, leg bones, leg muscles, and skin. All procedures were approved by the Local Committee for the Ethics of Animal Experimentation in Warsaw, the Voivodeship Chief Nature Conservator, and were compatible with the standards of the Polish Law on Experimenting on Animals, which implements the European Communities Council Directive.

Sediment samples were collected from a layer at a depth of 0–10 cm from three locations of the BUK pond where eggs and tadpoles were present. Concentrations of thallium (and additionally lead) were measured in sediments in order to assess the levels of xenobiotic metals in general.

### Chemical analysis

After the samples were collected, the spawn, juveniles, and the isolated skins of adult toads were washed with DI water. All biological samples were stored at −28 °C, and before the digestion, dried for 48 h at 80 °C; 250–300 mg of the samples were weighed into a polytetrafluoroethylene (PTFE, teflon) vessel, and digested with 2 ml 65 % HNO_3_ suprapure in a closed microwave system. After cooling down, the vessels were opened and 0.25 ml 30 % H_2_O_2_ was added. Then, the digestion procedure was continued for another 3 min. at 750 W. After digestion, the samples were diluted in 10 ml flasks and stored at 4 °C before analysis by ICP-MS. The sediments were dried (open air over 24 h, then in the laboratory dryer at 50 °C over 5 h), subsequently sieved (1 mm), and totally decomposed using a three-step digestion microwave-assisted procedure as described by Krasnodębska-Ostręga et al. (2005).

An inductively coupled plasma mass spectrometer SCIEX Elan 6100 (Perkin Elmer, USA) was used. The three points calibration curve method was applied to quantitative determination. The ICP-MS measurements in 25, 100, or 2500 time-diluted solutions after digestion were repeated at least three times. Blanks were included in the analysis.

Due to the lack of appropriate reference materials to evaluate the accuracy of measurements of the thallium concentration in animal samples, we used BCR White Cabbage-679 as our reference material. In all four reference samples, thallium concentrations were lower than detection limit = 0.005 (the expected value was 0.003 mg/kg d.w.).

### Statistical data

The analysis of variance ANOVA was used to compare the BUK and CHE samples. Differences between thallium concentration in the livers, kidneys, bones, muscles, and skins of adult toads from the BUK were assessed with Tukey’s test. Statistical calculation was performed with SAS 9.2 for Windows.

## Results and discussion

### Adult toads and individuals after metamorphosis

From among all tissues of the BUK adult toads that were collected, the livers showed the highest accumulation of thallium (mean 3.98 mg/kg d.w. and maximum value—18.63; Tukey’s test *p* < 0.05; Table 1). For as many as 28 livers out of 29 BUK-tested toads (96.6 %), concentrations reached values above 1.0 mgTl/kg d.w. which was treated as a sign of thallium contamination. Mean concentrations for the kidneys, muscles, bones, and skins were close to 1.0 mgTl/kg d.w., but great differences between individuals were noted, ranging from below the limit of detection to high values. The maximum detected concentrations of thallium were: for kidneys—4.69, bones—6.76, muscles—2.9, and skin—3.64 (Table 1).

**Table 1:** Thallium concentration (mg/kg d.w.) in the livers, kidneys, bones, muscles, and skin of adult toads as well as in the bodies of juveniles from the BUK and the CHE ponds

	BUK (polluted)			CHE (control)			
	*x* ± SD (range)	*N*	*n* (%)	*x* ± SD (range)	*N*	*n* (%)	ANOVA
Livers	3.98 ± 3.69^a^ (0.834–18.63)	29	28 (96.6 %)	0.27 ± 0.43 (<0.005–121)	20	1 (5 %)	*F* = 19.85 *p* < 0.0001
Kidneys	1.24 ± 1.57^bc^ (<0.025–4.69)	29	11 (37.9 %)	0.09 ± 0.30 (<0.005–1.34)	29	1 (5 %)	*F* = 7.57 *p* < 0.01
Bones	1.39 ± 1.40^b^ (<0.005–6.76)	29	17 (58.6 %)	0.03 ± 0.09 (<0.005–0.43)	20	0	*F* = 18.81 *p* < 0.001
Muscles	0.82 ± 0.80^c^ (<0.005–2.9)	27	9 (31.0 %)	0.02 ± 0.06 (<0.005–0.25)	20	0	*F* = 20.44 *p* < 0.0001
Skin	1.49 ± 1.18^b^ (<0.005–3.64)	14	8 (57.1 %)	0.01 ± 0.02 (<0.005–0.06)	11	0	
Juveniles	0.57 ± 0.78 (<0.005–1.79)	8		<0.005			

Superscript letters represent results of Tukey’s test (<0.05)

*N* number of studied individuals, *n (%)* number of individuals with Tl conc. >1 mg/kg d.w. and percent of total number of the samples, *x ± SD* mean value and standard deviation, *ANOVA* comparison of the BUK and CHE samples

Thallium concentrations in tissues of adult toads mating in the control CHE pond were significantly lower (Table 1—ANOVA results) as compared to the BUK pond. Mean values were at a level of about 0.0× mg/kg d.w. (typical for vertebrates from unpolluted regions), and the concentration of 1.0 mg/kg was exceeded in the liver and kidneys of only one individual.

The average accumulation of thallium in the carcasses of juveniles that went through their larval development and metamorphosis in the BUK pond after they spent over 7 weeks in the polluted water was only 0.57 mgTl/kg d.w. (max. 1.79; Table 1). Thallium was not detected in the CHE individuals of comparable age (<detection limit = 0.005 mg/kg).

The levels of thallium present in organs and internal tissues of adult toads (excluding the livers) were lower than those reported in other vertebrates living within a radius of 1–4 km from the smelting works in Bukowno. The accumulation of thallium in the livers of bank voles (*Myodes glareolus)* reached the level of 14.53 mg/kg, and in the kidneys 34.27 mg/kg, while the accumulation in the livers and kidneys of wood mice (*Apodemus sylvaticus*) was as high as 11.34 and 44.05, respectively (Dmowski et al. 1998). Cases of distinct decrease in the amount of hairs (allopecia), characteristic for thallium poisoning, were also noted. In another sub-population, concentration of Tl in livers was up to 49.57 mg/kg d.w. (Dmowski and Badurek 2001). Magpies (*Pica pica*) from the Bukowno area contained in their kidneys as much as 14–45 mgTl/kg d.w. (Dmowski et al. 1998).

Compared with data concerning the BUK toads, thallium levels found in wild vertebrates with symptoms of thallium poisoning were several dozen times higher, for example in carnivorous mammals (Clausen and Karlog 1974; Munch et al. 1974) or birds of prey (Cromartie et al. 1975; Clausen and Karlog 1977). Significantly higher concentrations of thallium were also observed in certain animals (dabbling ducks from Japan) that did not display any ill effects (Mochizuki et al. 2005). Data on thallium levels in tissues of amphibians from polluted regions are exceptionally scarce and they were not available for comparison.

Large amounts of Tl in sediments from the BUK pond (Table 2) and in neighboring soils suggested a possibility of much higher levels of thallium in tissues of toads. Toads that hibernate in the terrestrial holes close to water bodies in which they breed may absorb pollutants through their permeable skin and thus their tissues should accumulate much more thallium.

**Table 2:** Thallium and lead concentrations (mg/kg d.w.) in laid 1-day-old eggs from the BUK and CHE ponds, comparison to Tl and Pb levels in sediments of the BUK pond

	Laid 1-day-old eggs				Sediments	
*N*	Tl^a^ [mg/kg s.m.]		Pb^b^ [mg/kg s.m.]		Tl [mg/kg s.m.]	Pb [mg/kg s.m.]
	BUK	CHE	BUK	CHE	BUK	BUK
1	20.52	<0.005	3.49	0.59	8.423	5117.31
2	21.50	<0.005	2.09	0.57	6.740	4799.02
3	21.08	<0.005	2.05	0.39	7.479	4525.46
4	19.78	<0.005	2.90	0.35		
5	8.96	<0.005	1.35	0.65		
6	5.14	<0.005	3.14	0.79		
7	5.20	<0.005	1.40	3.80		
8	5.07	<0.005	4.62	0.77		
9	4.87	<0.005	3.98	0.05		
10	27.61	<0.005	1.80	1.61		
x ± SD	13.97 ± 8.90	0.005	2.68 ± 1.12	0.96 ± 1.08	7.55 ± 0.84	4813.93 ± 296.21
range	4.89–27.61		1.35–4.62	0.05–3.80		

Superscript letters represent ANOVA comparison of the BUK and CHE samples. For Tl in eggs *F* = 24.65; df = 1; *p* = 0.0001; for Pb in eggs *F* = 12.31; df = 1; *p* = 0.0025

*N* number of the sample, *x ± SD* mean value and standard deviation, *<0.005* detection limits for Tl

Adult common toads spend a relatively short time in direct contact with the aquatic environment, and individuals occupy different locations within their terrestrial habitat thus their exposure to contamination with thallium may vary. Such differences may be responsible for the wide spread of the individual results that have been recorded. Those individual common toads which readily feed on earthworms or live in areas where earthworms are especially numerous are at a particular risk for thallium accumulation, since earthworms tend to store large quantities of extremely polluted soil and litter in their digestive tracts. Toads also eat many *Limax* and *Arion* slugs that are known for their tendency to accumulate thallium (in hepatopancreas particularly). The slugs living within the BUK area contained up to 33.48 mgTl/kg d.w. (Dmowski and Badurek 2001).

Some authors believe that metamorphosed anurans emigrating from their birth ponds can transport large quantities of toxic metals to nearby terrestrial habitats (e.g., Roe et al. 2005; Snodgrass et al. 2003; Unrine et al. 2007). Most probably, this vector is not significant in the case of thallium.

The present research cannot tell much about the direct negative influence of thallium on the development and health of toads since the BUK pond was also contaminated with extreme amounts of Pb and Cd as well as Zn and other elements that are typically associated with Pb-Zn ores (Krasnodębska-Ostręga et al. 2005). However, the BUK toads show many characteristics that demonstrate their adaptation to this extremely adverse environment—most likely due to the selective pressure that was exerted on the consecutive generations that inhabited this area. For example, we were unable to detect any external developmental abnormalities in either adult individuals, tadpoles, or juveniles from both researched ponds.

### Spawn and oocytes

Very high quantities of thallium were found in the eggs that were deposited in the BUK pond (mean 13.97 mg/kg d.w; max. 27.61; Table 2). Concentrations that were much higher than 1 mg/kg d.w. were present in all samples. For half of the samples, Tl concentration was close to or exceeded 20 mg/kg d.w.

Thallium was missing from the egg masses collected in the control pond CHE (all samples below the limit of detection 0.005 mg/kg d.w.).

Several lines of evidence suggest that thallium in the spawn was not absorbed from water or sediments but was transferred directly from the bodies of the females. Firstly, the earlier preliminary examination of common frogs *Rana temporaria* from the BUK area demonstrated thallium accumulation in internal tissues in the range of 5.09–15.39 mg/kg d.w., while the level of thallium in oocytes of the ovary was 3–10 times higher—as much as 51.61 mg/kg d.w. (Dmowski et al. 2002). It is also safe to ignore absorption of Tl from water due to the very low concentration of thallium in the water of the BUK pond (0.070 ± 0.001 μg/L; Krasnodębska-Ostręga et al. 2005). Finally, the comparison of Tl and Pb concentrations in 1-day-old spawn demonstrates entirely different mechanism of accumulation of these metals (Table 2). Only a minimal amount of Pb was accumulated in eggs, in spite of huge lead concentrations in sediments (up to 5117.31 mg/kg d.w.), suggesting both an absence of transfer between females and spawn as well as between sediments and spawn. The deposition of Tl in sediments was very significant, yet its accumulation in eggs was 2–3-fold higher, although the spawn was collected for the analysis no later than 1 day after the oviposition. The mentioned data clearly speak of a possibility of maternal transfer.

The issue of maternal transfer of toxic elements to amphibian eggs was raised by several authors, for example by Hopkins et al. (2006) for strontium and selenium. Many authors have also suggested the maternal transfer of organic contaminants (e.g., Kadokami et al. 2004; Sparling 2010; Peng-Yan et al. 2011) and mercury (Bergeron et al. 2010, 2011) to frog and toad spawn. In case of very toxic lipophilic organic compounds and mercury, the maternal transfer may represent the most important route of exposure for amphibian embryos because concentrations of these xenobiotics in water are generally low. The transfer of selenium to eggs is likely due to its substitution for sulfur, and transfer of strontium results from the substitution for calcium (Hopkins et al. 2006). Thallium is likely to replace the potassium present in the oocytes. Sherstobitov et al. (2003, 2010) demonstrated that the transport of monovalent thallium across the membranes of lamprey oocytes can occur in at least two ways: through NA/K-pump as well as by the mechanism of Na, K, and Cl cotransport. Some authors have described the transfer of cadmium from the anuran females to eggs (Grillitsch and Chovanec 1995; Kotyzova and Sundeman 1998); others (e.g. Hopkins et al. 2006) did not report this phenomenon even in highly contaminated areas.

The accumulation of Tl in the egg masses may have an additional, important environmental impact. While certain authors claim that eggs of the common toad are unpalatable (Duellman and Trueb 1986), others disagree and believe that many predators use them as a good source of nourishment (e.g., Gunzburger and Travis 2005; Henrikson 1990). If the tendency to accumulate thallium in the spawn is common for amphibian species in general in the areas similar to the BUK site, gelatinous egg masses or strings may be a significant source of thallium for predators. Non-resident predators, for example water birds that fly over larger distances to the polluted water bodies in search of food, may be especially vulnerable since their sporadic visits to the polluted area do not allow for the development of adaptations to high concentrations of thallium.

The spawn can also be used as a sensitive indicator of thallium pollution and can provide information about the presence of this element in the environment. This method may potentially replace more invasive procedures such as dissection of tissues of adult individuals.

### Pond sediments

Mean concentration of 7.55 ± 0.84 mg Tl/kg d.w. was detected in three samples of bottom sediments that were collected from the BUK pond (Table 2). Very similar levels have been found in sediments during the largest environmental disaster associated with thallium poisoning (in a region adjacent to a cement works at Lengerich, Germany, 1979/80). Thallium concentration of 8.7 mgTl/kg d.w was detected in river sediments at a distance of 4 km from the plant, and 18 mg/kg d.w. was noted at a distance of 1 km from the plant (Brockhaus et al. 1981).

The results of the BUK sediment analysis point to a very high level of exposure to thallium (as well as lead; Table 2) of the local toad population. Very similar quantities of Tl and Pb were found in this pond in an earlier study by Krasnodębska-Ostręga et al. (2005). Such sediments may pose a substantial threat as a source of xenobiotics to adult individuals and to all developmental stages, as well as to the bottom-dwelling invertebrates that constitute the source of food to amphibians.
